# Regional Lean Soft Tissue and Intracellular Water Are Associated with Changes in Lower-Body Neuromuscular Performance: A Pilot Study in Elite Soccer Players

**DOI:** 10.3390/ejihpe12080064

**Published:** 2022-07-22

**Authors:** Tindaro Bongiovanni, Grant Tinsley, Giulia Martera, Carmine Orlandi, Federico Genovesi, Giuseppe Puleo, Alessio Rossi, Athos Trecroci

**Affiliations:** 1 Performance and Analytics Department, Parma Calcio 1913, 43044 Parma, Italy; tindaro.bongiovanni@gmail.com; 2 Department of Biomedical Sciences for Health, Università degli Studi di Milano, 20129 Milano, Italy; athos.trecroci@unimi.it; 3 Department of Kinesiology & Sport Management, Texas Tech University, Lubbock, TX 79409, USA; grant.tinsley@ttu.edu; 4 Nutrition Department, Spezia Calcio, 19123 La Spezia, Italy; giumartera@gmail.com; 5 Department of Sport Science, Tor Vergata University of Roma, 00133 Rome, Italy; carmine.orlandi@uniroma2.it; 6 Medical Department, Manchester City Football Club, Manchester 03101, UK; federico.genovesi@mancity.com; 7 Reggina 1914 Football Club, 89132 Reggio Calabria, Italy; gpuleooo@gmail.com; 8 Department of Computer Science, University of Pisa, 56126 Pisa, Italy; 9 National Research Council (CNR), Institute of Information Science and Technologies (ISTI), 56125 Pisa, Italy

**Keywords:** body composition, soccer, hydration, vertical jump, strength

## Abstract

The assessment of body composition over a competitive season provides valuable information that can help sports professionals to evaluate the efficacy of training and nutritional strategies, as well as monitoring athletes’ health status. The purpose of this study was to examine the association of changes in body composition and hydration status with changes in lower-body neuromuscular performance in soccer. Twenty-two male professional soccer players (mean ± SD; age: 26.4 ± 4.8 years; height: 184.3 ± 5.7 cm; body mass: 81.1 ± 6.5 kg; body fat: 11.6 ± 1.5%) took part in the study, for which they were tested at the initial and final stage of the competitive season. Total (whole body) and regional (arms and legs) lean soft tissue (LST) were estimated to obtain the body composition profile. Total body water (TBW) content, including extracellular (ECW) and intracellular (ICW) water, was obtained to monitor players’ hydration status. Countermovement jump (CMJ) height, power, and strength were used to derive players’ lower-body neuromuscular performance. The results showed that changes in legs LST and ICW significantly (*p* < 0.01) explained (r^2^ = 0.39) the improvements in CMJ height, power, and strength from the initial to the final stage of the season. Given the high demand imposed on the lower limbs during a soccer season, being more susceptible to change compared to whole-body LST, assessing regional LST and ICW would be more appropriate to provide extended information on players’ readiness.

## 1. Introduction

The assessment of body composition over a competitive season provides valuable information that can help sports professionals to evaluate the efficacy of training and nutritional strategies, as well as monitoring athletes’ health status [1]. Indeed, ongoing measurements can help to create a longitudinal anthropometric profile of each player [2] in the attempt to avoid variation in body composition (e.g., fat mass, fat-free mass, and body water) that would be detrimental to performance [3]. It is well-known that a gain in fat mass is linked to an increment in a player’s energy demands during training and competition and to an impairment in performance in terms of power and acceleration [4,5]. Moreover, an increase in lean soft tissue (LST) is found to be strongly correlated to an increment in strength and power performance [6,7]. Besides the assessment of the whole-body amount of LST, knowing its distribution among the body segments (e.g., arms and legs) appears relevant for monitoring potential adaptations from specific training regimens, evaluating potential injury risk, managing rehabilitation, and optimizing high-intensity actions such as sprinting, change of direction, and jumping [8]. Nowadays, these types of actions are crucial in high-intensity-level competitive soccer, so much so that their enhancement-associated factors are essential [9].

Previous studies reported that body composition and physical performance changes occur during the competitive season in volleyball [10,11], handball [12,13], basketball [14,15], rugby [6], and in soccer players [7,16]. However, to date, few studies have evaluated the impact of the change in body composition on physical performance within a competitive season. Colyer et al. [17] showed clear relationships between the changes in countermovement jump and leg press and the changes in fat-free mass (r ± 90% CI; 0.53 ± 0.26 and 0.35 ± 0.28, respectively) and fat mass (−0.44 ± 0.27 and −0.37 ± 0.28, respectively). Conversely, Suarez-Arrones [18] reported a reduction in absolute whole-body fat mass (−5.2%) and an increase in whole-body fat-free mass (+2.5%), and the author also reported an increase in arms LST from September to May in elite soccer players. Albeit favorable changes, authors found that these body composition improvements were not related with improvements in linear sprint and half-squat performances.

Given that the major part of skeletal muscle mass, a component of body composition related to force and power development, resides at the appendicular level (legs and arms), the LST of arms and legs can provide a good estimate of the total body skeletal muscle mass [19]. Not long ago, studies reported the validity of regional body composition output provided by some contemporary assessment methods. For example, estimates of the composition of the arms, legs, and trunk can add specificity to a body composition evaluation and could potentially have greater functional and health implications for morbidity and mortality [20,21]. In the sports setting, it was recently showed how lower-body phase angle (LPhA) is a useful indicator of lower-limb cellular integrity and muscle quality, and it is more informative than whole-body PhA for explaining sprinting and jumping performance in youth soccer players. Moreover, it was also found that an in-season increase in LPhA appeared to be associated with an increase in vertical jump in elite soccer players. Similarly, Honorato et al. [22] showed that changes in fat mass, fat-free mass, and regional PhA impacted the power and aerobic capacities of professional soccer players over a pre-season period. These preliminary results may encourage a regional approach as being more informative than a whole-body assessment to explain physical performance changes [23].

For a more comprehensive assessment, monitoring the total body water (TBW) and its constituents, i.e., extracellular (ECW) and intracellular water (ICW), may represent an additional and valuable approach to control for potential body composition changes linked to physical performance in athletes [24]. Of note, ICW has been shown to be moderately correlated with vertical jump height and power [24,25,26], and it is also considered a good predictor of lower-limb neuromuscular performance [24]. Considering the importance of body composition for soccer players’ readiness, its evaluation should take into account not only fat mass, fat-free mass, and LST (total and regional) but also ECW and ICW. These variables would make coaches and practitioners aware of the development of their players’ body composition throughout the competitive season, having the chance to adjust training programs and, possibly, prevent injuries and enhance sports performance.

In this study, we investigated whether the changes in body composition and body water were related to lower-body neuromuscular performance changes over the course of a competitive season. In soccer, given the high demand imposed on the lower limbs during a season, we hypothesized that regional (i.e., leg) LST would be more susceptible to a change in response to the entire competitive season period, as compared to other whole-body parameters (e.g., whole-body LST or fat-free mass).

## 2. Materials and Methods

### 2.1. Subject

Twenty-two male soccer players (mean ± SD; age: 26.4 ± 4.8 years; height: 184.3 ± 5.7 cm; body mass: 81.1 ± 6.5 kg; body fat: 11.6 ± 1.5%) playing for a professional Italian soccer team competing in the Serie A (season 2020–2021) volunteered to participate in this study. Inclusion criteria were: (1) being outfield players, (2) having a current professional contract with the club, (3) no history of febrile illness at the time of the initial and final assessment, (4) attending > 8 h training per week, and (5) no injury at the initial and final stage of assessments. Participants were excluded if they were goalkeepers, taller than 190 cm [27], or had previously undergone serious muscle injuries over the competitive season. The weekly training routine of each participant consisted of 5 sessions per week for an average duration of 80 min each (including endurance running, sprint running drills, technical exercises, and small and large side games) and an official match per week. All participants provided a signed informed consent prior to participating in the study. The study protocol was conducted according to the Declaration of Helsinki and was approved by the Ethics Committee of the University of Milan (approval number: 32/16).

### 2.2. Procedure

This observational study included two assessments at the beginning (from 14 to 20 September 2020) and at the end (from 17 to 23 May 2021) of the competitive season. Players’ body composition was recorded in the morning (from 8.30 a.m. to 9.30 a.m.) following a 12 h food and fluid fast and with the abstinence from caffeine or alcohol during the preceding 24 h. Likewise, the participants were requested to avoid vigorous exercising within 24 h before assessing. After the body composition recording, a standardized breakfast was consumed by participants as employed in the previous studies [28]. Following breakfast (i.e., 2 h after), participants performed a lower-body neuromuscular test (vertical jump).

### 2.3. Body Composition

Body weight and height were measured to the nearest 0.1 kg and 0.1 cm, respectively, via a mechanical stadiometer (Seca 769, Hamburg, Germany) while barefoot and wearing a bathing suit. Bone mineral content (BMC) and estimates of total and arm appendicular LST (ALST) and legs LST (LLST) and fat mass were obtained by Dual-Energy X-ray Absorptiometry on a Lunar Prodigy scanner (General Electric, Boston, MA, USA) with enCORE software (v. 17). Fat-free mass was also obtained by the sum of LST and bone mineral content. The scanner was calibrated daily prior to scanning, according to manufacturer indications using a calibration block. Participants’ positioning took place using custom-made foam blocks in order to promote reliability and standardization of measurements, as previously indicated by Nana et al. [27]. For soccer players that were too large to fit within Dual-Energy X-ray Absorptiometry dimensions, partial-body scans were performed, and excluded body portions were estimated via reflection scanning. This method has been shown to induce minimal error in DXA results [29,30]. For all reflection scans, the participant was positioned so that the entirety of the right side of the body fell within the scanning field. The enCORE software (v17, General Electric, Boston, MA, USA) was utilized for the segmental analysis of the arms, legs, and trunk. A trained physician manually adjusted the lines used to establish boundaries between body regions. For arms, the line was placed directly through the glenohumeral joint. All tissue distal to this line, for example, from the glenohumeral joint to the tips of the phalanges, was designed as the arm segment. For legs, the line was placed through the femoral neck, directly perpendicular to the axis, and angled such that the entirety of the pelvis remained in the trunk region. All tissue distal to this line, for example, from the femoral neck to the tips of the phalanges, was identified as the leg region.

Bioelectrical impedance analysis (BIA) was performed using a phase-sensitive bioelectrical analyzer (BIA 101 BIVA PRO, Akern, Florence, Italy). The device emits an alternating sinusoidal electric current of 250 μA at an operating monofrequency of 50 kHz (±0.1%). The device was calibrated using the standard control circuit supplied by the manufacturer that has a known impedance. Participants were positioned supine with a leg opening of 45° with respect to the midline of the body and with the upper limbs positioned 30° away from the trunk. After cleaning the skin with alcohol pads, four adhesive electrodes (Biatrodes Akern Srl, Florence, Italy) were placed on the back of the hands and four other electrodes on the neck of the corresponding feet, keeping a distance of 5 cm between each electrode [31]. The proximal hand electrode was positioned between the radial and ulnar styloid processes, directly superficial to the distal radioulnar joint. The distal hand electrode was positioned in the center of the third proximal phalanx. The proximal foot electrode was placed directly between the medial and lateral malleoli at the ankle. The distal foot electrode was placed immediately proximal to the second and third metatarsophalangeal joints.

From the raw BIA variables, estimates of TBW and ECW were obtained using equations (Equations (1) and (2)), specific for athletes by Matias et al. [32], where Wt refers to body mass (kg), sex is a binary value where 0 and 1 refer to female and male, respectively, S is the stature (cm), Rz reflects the resistance (ohm), and Xc is the reactance (ohm).

TBW (L) = 0.286 + 0.195 × S2/Rz + 0.385 × Wt + 5.086 × sex(1)

ECW (L) = 1.579 + 0.055 × S2/Rz + 0.127 × Wt + 0.006 × S2/Xc + 0.932 × sex(2)

ICW was then calculated by substructing ECW from TBW. Moreover, the length of vector (ZH) was obtained using the raw BIA parameters by the formula [33,34] as shown in Equation (3), where Rz, Xc, and S refer to resistance, reactance, and stature in cm, respectively.

ZH = √(Rz^2^ + Xc^2^)/(S/100) (3)

### 2.4. Hydratation Status

BIA device is highly reliant upon the body’s fluid content and it is important that hydration is standardized or controlled when utilizing it [35]. For this reason, urine-specific gravity (USG) has been determined to assure that athletes were in a hydrated state, defined as the value of urine-specific gravity ≤ 1.020 [36,37]. Specific gravity of the first morning urine collected in both the first and second time point was assessed within 30 min of collection using a clinical hand-held refractometer (ATAGO Co., Tokyo, Japan).

### 2.5. Dietary Intake

Participants were asked to complete by hand their three 24 h food records, corresponding to the assessment weeks (during the first and last weeks of the season). For this purpose, a trained nutritionist provided detailed oral and written instructions. Participants were instructed to describe the foods, fluids consumed, estimate the amounts using standardized household measures or record the weight or volume, and annotate the commercial name of packaged food. As an alternative, players were asked to send photos of the consumed meals. Data obtained from the food records were transformed into energy and nutrients using a dedicated software (WinFood Pro 3.4, Medimatica, Colonnella, Teramo, Italy). Estimated daily energy and nutrients intake are presented in Table 1.

### 2.6. Physical Tests

Each participant executed three countermovement jumps (CMJs) trials on a portable force platform (Quattro Jump, Kistler, Winterthur, Switzerland). Each trial was performed from a standing position with hands placed on the hips. During an individual trial, the participants quickly bent downward and then performed a fast upward push to reach the highest height from the lower-limbs action. A recovery of 2 min was allowed between trials. CMJ height, power, and strength were recorded within the upward phase by a dedicated software (Kistler software version 1.1.1.4, Kistler, Winterthur, Switzerland). The highest jump trial was used in the analysis.

### 2.7. Statistical Analysis

Paired t-test or Wilcoxon signed-rank test were performed in order to evaluate the difference between the initial and the final stage of the competitive season in accordance with the normality of data distribution assessed by Shapiro–Wilks’ Normality test. The Cohen’s d effect size was calculated, and threshold values were d < 0.2 (trivial), 0.2 < d < 0.5 (small), 0.5 < d < 0.8 (moderate), and d > 0.8 (large). Data are presented as mean ± SD. Relative changes were expressed as means and confidence intervals (CI). Moreover, correlation analyses between CMJ (i.e., height, power, and strength) and body composition parameters were assessed by Spearman’s rank correlation coefficient. Stepwise forward linear regression was performed in order to explain the variance of CMJ parameters based on body composition parameters. Forward selection is a type of stepwise regression which begins with an empty model and adds in variables one by one (from the most to the lowest statistically significant correlated variables). If the model goodness statistically significantly increases by adding an independent variable, this variable will be maintained in the model; otherwise, it will be deleted. All the analyses were conducted by using Python 3.8 language programming. The statistical significance was set at *p* < 0.05.

## 3. Results

Table 2 shows the descriptive statistics of all the variables included in this study. Moreover, the statistical differences between the initial and the final stage of the competitive season were also reported. As observed in Table 2, all the CMJ parameters show a statistically significant increment in the final part of the competitive season. Similarly, the BMC, fat mass, LST, ALST, LLST showed statistically significant changes between the initial and final stage of the season. Whereas, among the BIA parameters’ raw (i.e., Rz, Xc, and PhA) and derived metrics (i.e., TBW, ECW, and ICW), only the changes for the ICW, ECW, and TBW were statistically significant between the two seasonal time points.

**Figure 1:**
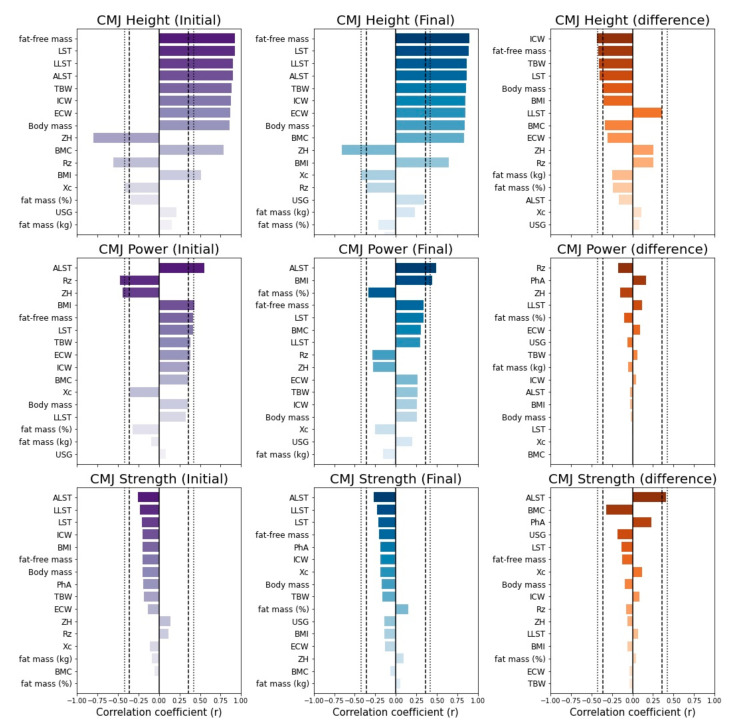
Barplots of the correlation coefficient between CMJ and body composition parameters. Darker colors indicate stronger correlations. Dashed and dotted lines indicate a statistical significance of about 0.10 and 0.05, respectively. CMJ, countermovement jump; BMI, body mass index; BMC, bone mineral content; FM, fat mass; FFM, fat-free mass; LST, whole-body lean soft tissue; ALST, arms lean soft tissue; LLST, legs lean soft tissue; TBW, total body water; ICW, intracellular water; ECW, extracellular water; USG, urine-specific gravity; Rz, resistance; Xc, reactance; PhA, phase angle; ZH, length of vector.

Figure 1 shows the correlation between the CMJ parameters and the body composition parameters at both the initial and final stage of competition, and the difference between the two seasonal time points. Almost all the body composition parameters were strongly related with CMJ height. ALST and BMI significantly correlated with CMJ power at both the beginning and end of the competitive season. Moreover, Rz and ZH were negatively correlated with CMJ power only at the final stage. No statistical correlation was found between CMJ strength and the body composition parameters. The change in ICW was moderately correlated with the change in CMJ height (*p* < 0.05, Figure 1). Moreover, the changes in fat-free mass, TBW, LST, body mass, and LLST show small-moderate correlations with the change in CMJ height (*p* < 0.10), Figure 1). ALST demonstrated a small-moderate relationship with CMJ strength (Figure 1). No statistically significant correlations were detected between the changes in the raw BIA parameters and the changes in CMJ power (Figure 1).

Table 3 and Table 4 show the results of the stepwise forward linear regression. As regards CMJ height, 39% of its variance is explained by the change in LLST (coefficient = 0.27 ± 0.10, *p* = 0.02) and ICW (coefficient = −0.49 ± 0.20, *p* = 0.02). In contrast, the changes in ALST (coefficient = 41.31 ± 14.74, *p* = 0.01) and BMI (coefficient = −27.30 ± 16.84, *p* = 0.05) explained 31% of the change in CMJ strength. No statistically significant models significantly explained the change in CMJ power by the change in the BIA parameters.

## 4. Discussion

The main aims of this study are to investigate the changes in body composition and body water and their association with lower-body neuromuscular performance. The main findings of this study were that changes in ICW and legs LST explained almost 40% of the improvements in CMJ height (r^2^ = 0.39; *p* < 0.01), confirming our previous hypothesis that regional (i.e., leg) LST would be more susceptible to change during the competitive season period. The fact that we found a link between CMJ height improvements and ICW changes appears interesting. Although such a relationship would need further investigations, it might be explained by “the cell swelling theory” originally described by Haussinger et al. and Lang et al. [38,39]. They affirm that intracellular volume is a pivotal signal for the metabolic adjustment of cell metabolism, as cellular swelling drives to anabolism while cellular shrinkage to catabolism [38]. An enhanced cellular hydration (i.e., ICW increases) could reflect an increase in glycogen storage (due to the great osmotic power of glycogen itself) that might act as an anabolic signal by cellular swelling. According to Silva et al. [24], ICW may stimulate pathways that increase protein synthesis. This would have put the players in a condition to have benefit from such an anabolic signal under a muscular function point of view (e.g., strength levels), as observable in the CMJ changes. Indeed, an association between ICW with upper-body power and maximal strength has been found in individual [40,41] and team sport athletes [24].

In the current study, ALST and BMI are able to describe CMJ strength enhancement (r^2^ = 0.31; *p* < 0.03). The lower decrement in ALST and BMI induced a gain in CMJ strength. This outcome indicates that the players remarkably reducing their ALST showed a marked decrease in CMJ strength over the season, whereas those who roughly kept a constant BMI (low increment) showed a higher increment in CMJ strength. These results are in line with our previous study [42] in which high performers in a vertical jump test presented a higher BMI (23.9 ± 1.25 vs. 22.8 ± 1.10 kg/m^2^; *p* = 0.03, ES = −0.91) and corrected arm muscle area (59.39 ± 6.64 vs. 50.74 ± 6.82 cm^2^), which is already considered a predictor of sprint performance in young soccer players [28]. This is not surprising as CMJ has also been found to be directly correlated with 10 m and 20 m sprint performances [43,44] and relative strength during dynamic 1 RM squat and power [45]. Accordingly, Suarez-Arrones et al. [18] found similar results in a study with elite professional soccer players of the Italian Serie A. Their results showed that ALST increased (7.6%) over the entire competitive period, suggesting that upper-body strength may have affected performance by concurring to both vertical and horizontal propulsive forces in sprinting and jumping.

To the best of the author’s knowledge, only a few studies have explored both the within-seasonal change in jump performance and body composition in soccer players [46,47]. Peart et al. [47] investigated the changes in the anthropometry, body composition, and physical fitness of female soccer players throughout a long period. The authors showed a significant decrease in fat mass while no remarkable changes were observed in fat-free mass at the end of the competitive season, which also reflect the CMJ height profile that did not change. Conversely, we found both an increase in fat-free mass and CMJ performance and a decrease in fat mass throughout the season. The different competitive level and gender may explain such a finding compared to the study of Peart and colleagues [47]. Moreover, we reported the TBW distribution that could also explain the changes observed in fat-free mass. Of note, Fessi et al. [46] showed CMJ (with no arms swing) improvements, while no significant changes were observed in fat mass and fat-free mass during the competitive season from July to December. This finding does not take into account the regional body composition that we found being more informative regardless of whole-body parameters, such as fat mass and fat-free mass. Additionally, it is worth noticing that Fessi et al. [46] considered a lesser period of investigation (i.e., six months), which may have affected the potential concurrent improvement of lower-limb neuromuscular performance and body composition.

Even though the present sample size was in line with previous studies in team sport athletes [48,49], its small size limits the interpretation of the results as well as their generalization. Further studies with a larger sample are warranted. Of note, our data are limited to male outfield professional soccer players, with no data on goalkeepers and elite female soccer players, which would have provided additional and extended knowledge on the association between regional and lower-limb neuromuscular performance. In keeping with this, future studies will have to be directed toward developing and validating biomarkers of body composition in the elite team sport population.

## 5. Conclusions

In the current study, ICW and regional LST were the best predictors for CMJ performance improvements over an entire competitive season in professional soccer players. This finding supports that assessing regional body composition would be more informative for monitoring lower-limb neuromuscular changes over the competitive season. The current preliminary results would encourage practitioners, coaching staff, sport scientists, and sport nutritionists to employ CMJ measures while also assessing their athletes by tracking ICW and regional LST to derive quantitative and qualitative information of the training status and inherent characteristics of athletes. For instance, they could help to identify individual strengths and weaknesses of physical performance (e.g., jumping tasks) while enabling more targeted, appropriate, and realistic training plans.

## Figures and Tables

**Table 1:** Average daily energy and nutrients intake.

Macronutrients	Mean ± SD
Energy (kcal)	3172.0 ± 210
Proteins (g)	149.0 ± 8.0
Proteins (g/kg)	1.8 ± 1.0
Total energy from proteins (%)	18.8 ± 2.2
Fats (g)	85.5 ± 6.0
Fats (g/kg)	1.04 ± 0.5
Total energy from fats (%)	24.2 ± 1.5
Carbohydrates (g)	408.8 ± 25.0
Carbohydrates (g/kg)	5.0 ± 1.0
Total energy from carbohydrates (%)	57.0 ± 3.0
Fiber (g)	40.0 ± 5.0

**Table 2:** This table provides the value of each parameter at the initial and the final stage of the competitive season. Moreover, mean difference, percentage of change, and statistical comparison are provided.

Features	Initial Stage of the Competitive Season	Final Stage of the Competitive Season	Difference [CI_low_, CI_high_]	Change (%)	ES
Body mass (kg)	81.2 ± 6.7	82.5 ± 6.5	1.82 [1.11, 2.53] ***	2.18 ± 1.8	0.28
BMI (kg/m2)	23.8 ± 1.0	24.2 ± 0.9	0.53 [0.33, 0.73] ***	2.18 ± 1.8	0.53
# BMC (kg)	3.84 ± 0.35	3.89 ± 0.36	0.09 [0.03, 0.14] ***	2.11 ± 2.9	0.23
# fat mass (kg)	9.98 ± 1.80	9.68 ± 2.03	−0.07 [−0.42, 0.28]	−1.34 ± 7.9	−0.05
# fat mass (%)	12.25 ± 1.79	11.67 ± 1.98	−0.36 [−0.72, −0.00] *	−3.53 ± 6.8	−0.24
# fat-free mass (kg)	71.25 ± 5.74	72.80 ± 5.23	1.89 [1.39, 2.40] ***	2.59 ± 1.4	0.33
# LST (kg)	67.41 ± 5.47	68.91 ± 4.94	1.80 [1.33, 2.28] ***	2.61 ± 1.4	0.33
# ALST (kg)	8.65 ± 1.14	8.41 ± 1.11	−0.10 [−0.33, 0.12]	−1.38 ± 6.2	−0.10
# LLST (kg) a	27.83 ± 2.99	28.98 ± 2.42	1.15 [0.70, 1.61] ***	4.19 ± 4.1	0.39
TBW (L)	51.04 ± 3.79	51.45 ± 3.44	0.69 [0.28, 1.10] **	1.34 ± 1.7	0.19
ICW (L)	30.98 ± 2.25	31.30 ± 2.04	0.48 [0.23, 0.72] ***	1.54 ± 1.7	0.21
ECW (L)	20.07 ± 1.55	20.16 ± 1.43	0.21 [0.03, 0.39] *	1.06 ± 1.2	0.14
USG a	1.020 ± 0.002	1.019 ± 0.003	−0.001 [−0.00, −0.00] ***	−0.13 ± 0.2	−0.53
Rz (ohm)	465.27 ± 26.25	467.09 ± 28.63	0.09 [−7.82, 8.00]	−0.04 ± 3.7	0.00
Xc (ohm)	65.09 ± 5.07	66.09 ± 5.13	0.52 [−1.08, 2.12]	0.62 ± 5.3	0.10
PhA (°) a	8.01 ± 0.69	8.11 ± 0.58	0.08 [−0.07, 0.24]	0.92 ± 4.1	0.11
CMJ Height (cm)	50.51 ± 4.49	52.50 ± 4.57	2.05 [1.78, 2.31] ***	3.91 ± 1.17	0.45
CMJ Power (W) a	4927.73 ± 451.6	4955.45 ± 420.7	36.00 [0.89, 71.11] *	0.78 ± 1.5	0.09
CMJ Strength (N)	2212.91 ± 250.6	2248.09 ± 235.4	47.77 [31.03, 64.52] ***	2.22 ± 1.8	0.21
ZH (ohm/m)	254.26 ± 17.32	255.17 ± 16.55	−0.02 [−4.32, 4.29]	−0.04 ± 3.65	0.00

Statistical significance: * *p* < 0.05; ** *p* < 0.01; *** *p* < 0.001. ^a^ refers to Wilcoxon signed-rank test. CI and ES indicate confidence interval and effect size, respectively. BMI, body mass index; BMC, bone mineral content; FM, fat mass; FFM, fat-free mass; LST, whole-body lean soft tissue; ALST, arms lean soft tissue; LLST, legs lean soft tissue; TBW, total body water; ICW, intracellular water; ECW, extracellular water; USG, urine-specific gravity; Rz, resistance; Xc, reactance; PhA, phase angle; CMJ, countermovement jump. ZH, length of vector. # DXA-measured parameters.

**Table 3:** Stepwise linear regression for CMJ height delta.

Feature	Coeff	Coeff Error	*p*-Value	CI Lower	CI Upper
Intercept	1.97	0.18	<0.001	1.60	2.35
ΔLLST	0.27	0.10	0.02	0.05	0.49
ΔICW	−0.49	0.20	0.02	−0.90	−0.09

Dependent variable: delta CMJ height; model r^2^ = 0.39 (*p* < 0.01). Δ LLST, delta legs lean soft tissue; ΔICW, delta intracellular water; coeff, coefficient.

**Table 4:** Stepwise linear regression for CMJ strength delta.

Feature	Coeff	Coeff Error	*p*-Value	CI Lower	CI Upper
Intercept	66.50	11.79	<0.001	41.83	91.18
ΔALST	41.31	14.74	0.01	10.46	72.16
ΔBMI	−27.30	16.84	0.05	−62.54	−7.93

Dependent variable: delta CMJ strength; model r^2^ = 0.31 (*p* = 0.03). ΔALST, delta arms lean soft tissue; ΔBMI, delta body mass index; coeff, coefficient.

## Data Availability

All the relevant data were provided throughout the text.
